# Adherence to customized vestibular rehabilitation therapy: influencing factors and clinical implications in vestibulopathy

**DOI:** 10.3389/fneur.2025.1538989

**Published:** 2025-02-05

**Authors:** Yeon Hee Im, Hyun Jin Lee, Eun-Ju Jeon

**Affiliations:** Department of Otorhinolaryngology-Head and Neck Surgery, Incheon St. Mary’s Hospital, College of Medicine, The Catholic University of Korea, Incheon, Republic of Korea

**Keywords:** vestibular disorders, vestibular neuritis, bilateral vestibulopathy, rehabilitation, patient adherence

## Abstract

**Objective:**

Customized vestibular rehabilitation therapy (CVRT) is an effective treatment approach for various vestibular disorders. However, low adherence significantly limits its efficacy, and factors influencing adherence remain underexplored. This study aimed to identify factors affecting adherence to CVRT across major vestibulopathy categories, including acute unilateral vestibular hypofunction (AUVH), chronic unilateral vestibular hypofunction (CUVH), and bilateral vestibular hypofunction (BVH).

**Methods:**

A retrospective analysis of 90 patients who were prescribed four sessions of CVRT and underwent the therapy was conducted. Patients were classified as adherent (≥3 sessions) or non-adherent (≤2 sessions). Demographic characteristics, baseline questionnaire scores, and vestibular function test (VFT) results were compared. Change in questionnaire scores and VFT results before and after CVRT, as well as mid-treatment follow-up questionnaire scores were analyzed.

**Results:**

Adherence rates were highest in CUVH (86.7%) and lowest in BVH (46.2%). Among patients with AUVH, poor functional reach test scores were significantly associated with lower adherence (*p* = 0.045). In the CUVH category, patients with mild dizziness in the initial questionnaire were non-adherent (*p* = 0.019). CVRT improved subjective dizziness symptoms and VFT parameters, with the greatest gains observed in AUVH. However, no significant differences in symptom improvement were found between adherent and non-adherent patients. Patients whose symptoms improved rapidly to a mild degree after starting the CVRT were more likely to be non-adherent, with this tendency being especially pronounced in those with AUVH.

**Conclusion:**

Adherence to CVRT varies by vestibulopathy category and is influenced by baseline symptom severity. Tailoring CVRT strategies based on individual clinical profiles may enhance adherence and optimize therapeutic outcomes.

## 1 Introduction

Vestibular rehabilitation therapy (VRT) is an effective treatment strategy to improve symptoms and quality of life in various disorders related to dizziness and imbalance (1–3). The primary mechanisms underlying VRT involve activation of vestibular compensation processes, such as vestibular adaptation, habituation, and sensory substitution, using neuroplasticity (4). VRT alleviates dizziness, enhances postural control, reduces oscillopsia, and mitigates psychological and emotional distress (5, 6).

Low adherence to VRT is recognized as a critical issue hindering the effectiveness of the treatment. An earlier study reported that less than half of the patients completed the recommended VRT programs (7). Factors such as limited accessibility, high costs, and temporary exacerbation of dizziness during exercises are also suggested as contributing factors to reduced adherence (8, 9). Higher adherence to VRT leads to greater effectiveness in relieving dizziness, improving mental stability, and enhancing daily functioning (8). Various attempts have been made to improve adherence to VRT, including brochures, internet platforms, virtual reality, and smartphone gaming systems (7, 10–12). Despite these advances, research specifically analyzing adherence factors in VRT remains few.

Customized vestibular rehabilitation therapy (CVRT) tailors exercises to the specific symptoms and functional impairments of individual patients (13, 14). Previous studies have shown that CVRT is effective in improving dizziness-related outcomes (15–17). However, CVRT poses substantial challenges for both patients and healthcare providers. From the patient’s perspective, CVRT requires frequent clinic visits, which can be time-consuming, costly, and particularly burdensome for individuals with mobility issues due to dizziness and balance disorders. CVRT poses significant challenges for healthcare providers, requiring the development of individualized exercise programs and substantial investments in personnel, time, and facilities.

Despite these efforts, the success of CVRT heavily depends on patient adherence. Non-compliance can undermine therapeutic outcomes, wasting the efforts of both patients and providers. Identifying and considering adherence factors in patient selection and program management is crucial for optimizing the efficiency and efficacy of this therapy. This study aims to identify factors influencing CVRT adherence across common vestibulopathy categories, with the goal of improving adherence and optimizing therapeutic outcomes.

## 2 Materials and methods

### 2.1 Patients and study design

**Figure 1:**
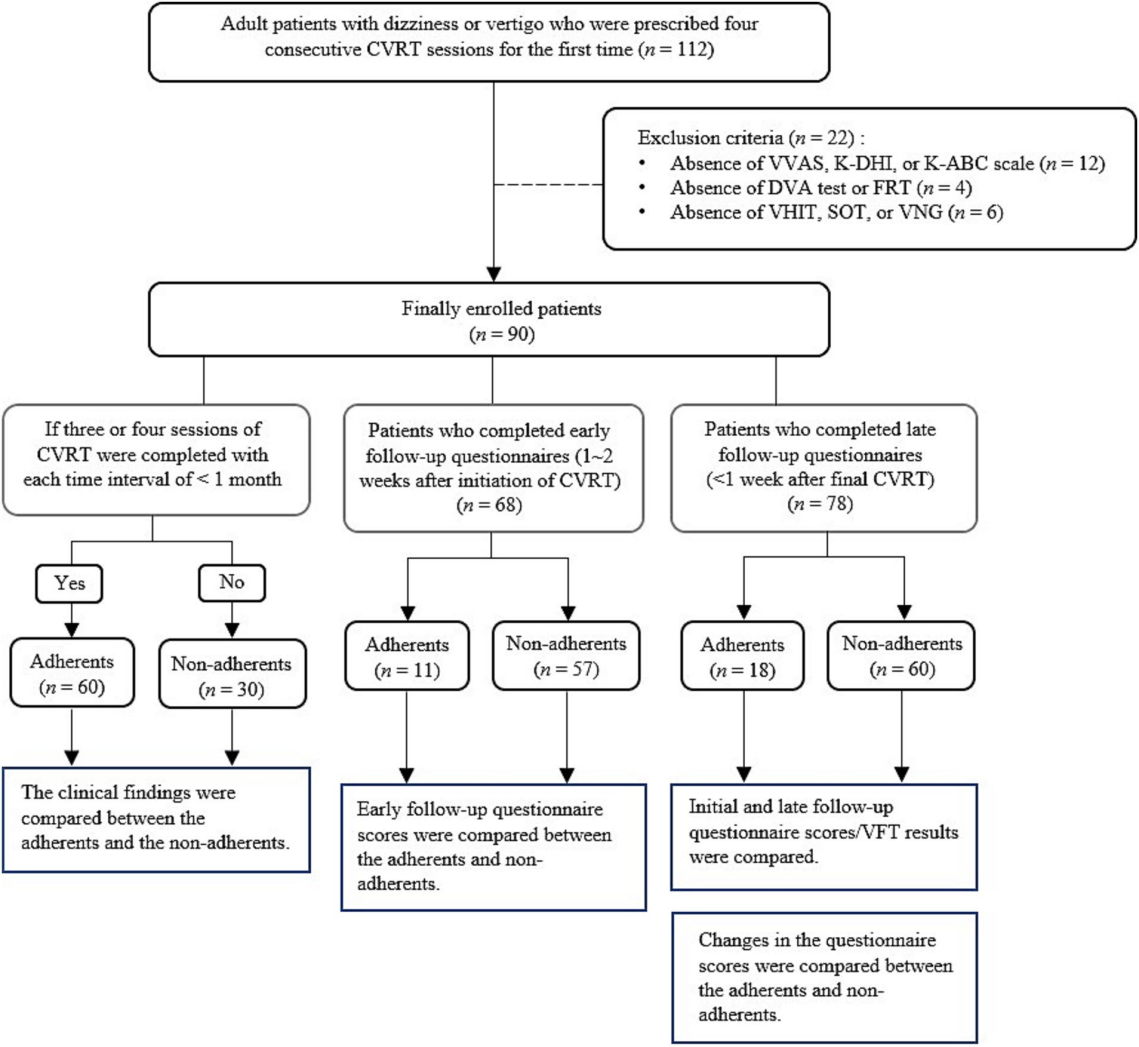
Study flow diagram. CVRT, customized vestibular rehabilitation therapy; VVAS, vestibular visual analog scale; K-DHI, Korean dizziness handicap inventory; K-ABC, Korean activities-specific balance confidence; DVA, dynamic visual acuity; FRT, functional reach test; VHIT, video head impulse test; SOT, sensory organization test; VNG, videonystagmography.

The retrospective chart analysis was conducted on patients who underwent vestibular rehabilitation therapy at Incheon St. Mary’s Hospital from 2017 to 2021. The Institutional Review Board at Incheon St. Mary’s Hospital approved the study in 2022 (approval number OC22RASI0129) and waived the informed consent process. The study process is illustrated in Figure 1.

All patients presenting with dizziness underwent thorough medical history-taking, physical examination, and vestibular function assessment. Those deemed to require CVRT were recommended four sessions of customized vestibular rehabilitation. The participants in CVRT were assessed for their subjective dizziness levels both before each treatment session and after completing the fourth treatment using the following questionnaires: Vestibular Visual Analog Scale (VVAS), Korean Activity-Specific Balance Confidence (K-ABC) Scale, and Korean Dizziness Handicap Inventory (K-DHI). For objective evaluation, the following vestibular function tests were conducted before the treatment started: Dynamic Visual Acuity (DVA) test, Functional Reach Test (FRT), Video Head Impulse Test (VHIT), Sensory Organization Test (SOT), and Videonystagmography (VNG).

While the majority completed the four sessions, some patients participated in only part of the sessions and discontinued treatment midway. To identify factors associated with treatment adherence, this study was conducted on patients prescribed four sessions of CVRT. Patients were excluded from the study if they missed any part of the questionnaire, physical examination, or balance test to be analyzed. Ultimately, a total of 90 patients were included in the study. Patients who completed at least three of the four CVRTs were classified as adherents, and those who completed only one or two were classified as non-adherents. This adherence classification was applied across the total study population and within each diagnostic category (CUVH, AUVH, BVH). For clarity, the adherent and the non-adherent subgroups within each diagnostic category were labeled as CUVH-adherents (CUVH-A), CUVH-non-adherents (CUVH-NA), AUVH-A, AUVH-NA, BVH-A, and BVH-NA, respectively. Each diagnostic category contains distinct numbers of adherent and non-adherent patients (Figure 1).

The study initially analyzed the demographic characteristics and diagnoses of the study population. A comparison was made between adherents and non-adherents within the total study population in terms of demographic characteristics, dizziness-related questionnaires, physical examination findings, and vestibular function test (VFT) results. Clinical findings were compared additionally between the adherent and non-adherent subgroups within each of the three prevalent disease categories (refer to 2.5 Diagnosis of vestibular disorders).

To examine the impact of symptoms changes on adherence, an additional analysis was performed on the 78 subjects who completed follow-up dizziness-related questionnaires within one week after the final CVRT session. For these 78 patients, study examined the presence of significant changes in questionnaire scores and VFT results before and after CVRT, as well as differences in symptom changes between adherents and non-adherents. Furthermore, among the 68 patients who completed the interim questionnaires between 7 and 14 days following the start of CVRT, the scores were compared between the adherents and non-adherents.

### 2.2 Dizziness-related questionnaires

#### 2.2.1 VVAS

The VVAS is used to quantify the severity of dizziness, oscillopsia, and imbalance experienced by patients. Each symptom was rated on a scale of 0 to 10, where 0 represents the absence of symptoms, and 10 represents the highest possible intensity of symptoms reported by the patient (18).

#### 2.2.2 K-DHI

The DHI questionnaire is designed to assess the challenges due to dizziness that individuals may face in their daily lives. It comprises 25 questions categorized into emotional, functional, and physical aspects. Each item was scored as 0, 2, or 4 points based on the presence and frequency of symptoms. The total score on the DHI ranged from 0 to 100, with higher scores indicating more severe symptoms (19). For patients of our study, the DHI questionnaires translated into Korean and validated by the Korean Balance Society was used (20). The minimally clinical important difference (MCID) value for the DHI was set at 18 points (19).

#### 2.2.3 K-ABC scale

The ABC scale measures patients’ confidence levels in performing 16 common body movements daily. Each movement was rated as a percentage, reflecting the patients’ perceived confidence in executing the movement. The average of these percentage scores was calculated as the overall score on the ABC scale (21, 22). The validated Korean version of ABC scale developed by the Korean Balance Society was used for the patients of this study (20). The MCID value for the ABC scale was set at 18.1% (23).

### 2.3 Physical examination

#### 2.3.1 DVA test

Dr. Hahn’s standard 3-m vision test chart was used to perform the DVA test. First, the patient stood at a distance of 3 m and their static visual acuity was assessed. Then, the patient’s DVA was measured while their head was actively rotated at a speed of 2 Hz with an amplitude of 20° to the left and right (24). The number of lines between static and DVA was recorded as the measurement result.

#### 2.3.2 FRT

In the FRT test, the patient was instructed to stand upright and extend their arms forward while making fists with their palms facing downward. The horizontal distance between the body and the third metacarpals was measured. Next, the patient was asked to bend their upper body forward as much as possible while keeping their arms parallel to the ground, and the distance between the body and the third metacarpals was measured again. The test was performed thrice and the average difference between the two distances was calculated to determine the result (25).

### 2.4 VFT

#### 2.4.1 VNG

Eye movements were recorded and nystagmus was measured using the Visual Eyes VNG System (Micromedical Technologies, Chatham, IL, USA). The caloric test was conducted by irrigating the ear with water or air. Caloric paresis (CP), a measure of asymmetry in response to caloric stimulation, was calculated using Jongkees’ formula: CP = [(left warm + left cool) - (right warm + right cool)] / [(left warm + left cool) + (right warm + right cool)] × 100% (26). The peak slow component velocity of each nystagmus was inputted into the formula for each term of measurement. CP of >25% or the sum of bithermal peak slow component velocities on each side <6°/sec was considered pathological (27, 28).

#### 2.4.2 VHIT

The patient was equipped with VHIT goggles (ICS Impulse; Natus Medical, Pleasanton, CA, USA) and instructed to maintain a forward gaze. An experienced examiner then rapidly and repeatedly rotated the patient’s head in three planes (left–right, right-anterior–left-posterior, and left-anterior–right-posterior) at an approximate range of 10–20°. The gain in vestibuloocular reflex (VOR) was determined by automatically calculating the relationship between the area under the eye velocity curve and the head velocity curve from the beginning of head movement until the velocity returned to zero (29). In VHIT, a VOR gain above 0.8 is often classified as normal, although variability exists in defining normal versus pathological values (30). In this study, a VOR gain of 0.8 was used as the cutoff to determine normal or abnormal function. For classifying specific subtypes of vestibular disorders, more detailed VHIT criteria were applied, as further explained in ‘*Section 2.5 Diagnosis of vestibular disorders*’.

#### 2.4.3 SOT

A SMART Balance Master (NeuroCom International Inc., Clackamas, OR, USA) was used to perform sensory organization test under six conditions. These conditions included: (1) eyes open with a fixed platform, (2) eyes closed with a fixed platform, (3) moving visual surround with a fixed platform, (4) eyes open with a moving platform, (5) eyes closed with a moving platform, and (6) moving visual surround with a moving platform. Each condition consisted of three 20-s trials. A composite score was automatically derived from all equilibrium scores obtained in each condition. The total number of falls during the 18 tests was also recorded. Any score or sensory ratio (e.g., somatosensory, visual, vestibular, and visual preference) below the 5th percentile of a reference database of individuals of the same age was defined as abnormal (31, 32).

### 2.5 Diagnosis of vestibular disorders

Diagnosis of the most vestibular disorders in this study were conducted following the International Classification of Vestibular Disorders, which were developed by the Classification Committee of the Bárány Society (CCBS). Acute unilateral vestibular hypofunction (AUVH) category included patients who satisfied CCBS-defined criteria for ‘acute unilateral vestibulopathy’. VOR function was deemed diminished, indicated by a VHIT gain below 0.7 or a CP of 25% or higher (27). The study noted the absence of specific CCBS criteria for chronic unilateral vestibulopathy. The diagnosis of chronic unilateral vestibular hypofunction (CUVH) in this research was based on a CP of 25% or higher with dizziness lasting beyond three months (33). Bilateral vestibular hypofunction (BVH) was diagnosed based on the criteria outlined by the CCBS, which include symptoms consistent with BVH and either a horizontal angular VOR gain <0.6 on VHIT or a reduced caloric response (sum of bithermal peak slow component velocities on each side <6°/sec) (28). Ménière’s disease category was assigned to those who matched the ‘definite Ménière’s disease’ or ‘probable Ménière’s disease’ criteria as per CCBS (34). Diagnoses for presbyvestibulopathy, persistent postural-perceptual dizziness, and vestibular paroxysmia were made following the respective CCBS criteria, with reduced VOR function ascertained through VHIT or caloric test for presbyvestibulopathy patients (35–37). Furthermore, individuals who fulfilled the criteria for ‘vestibular migraine’ or ‘probable vestibular migraine’ as established by the CCBS were placed into the vestibular migraine category (38).

### 2.6 Customized vestibular rehabilitation therapy

Customized vestibular exercises were prescribed for patients whose dizziness was triggered by head movements or postural changes. CVRT was performed under the supervision of a therapist trained in the basic knowledge of vestibular anatomy and physiology, vestibular diseases, and vestibular rehabilitation techniques. Customized vestibular exercises were performed one-to-one in 30–40-min sessions once a week.

Based on the patient’s symptoms and related disability, gaze stability exercises, balance and gait training, and/or habituation exercises were prescribed. Gaze stability exercises involved controlled head movements from side to side or up and down while focusing on a specific target. Initially, these exercises were performed in a seated position with slow head movements. The difficulty level was progressively increased by transitioning to standing or walking positions, accelerating head movements, or performing exercises on an unstable foam surface.

Vestibular rehabilitation for postural control involves training individuals to maintain a steady posture for a certain period while altering three sensory inputs necessary for postural balance: vestibular sensation, vision, and proprioception. Various methods are employed to manipulate sensory inputs by decreasing, blocking, or confusing sensations, or by disturbing sensory signals to induce changes. Postural control was initially started by having the patient stand on a fixed hard floor in the Romberg position with their eyes open for 30 s. Depending on the patient’s balancing function, the difficulty was gradually increased by altering sensory conditions, such as reducing visual or proprioceptive input. Gradual progression included tasks such as closing the eyes, narrowing the support base by transitioning to a sharpened Romberg position, standing on a dense foam mattress, or incorporating head movements or cognitive tasks.

Similarly, gait training involves walking a certain distance regularly every day, starting with shorter distances and gradually increasing to longer distances as individuals become accustomed. Like postural training, gait training involves varying and combining the three sensory conditions to gradually increase difficulty levels. If specific postures, movements, or situations triggered dizziness, habituation exercises were incorporated, exposing patients repeatedly to those provocative stimuli (33).

Patients were advised to receive supervised instruction and training once a week for four sessions. They were provided with printed instructions to perform the exercises learned during the sessions at home three times daily, for at least 10 min per session. The total recommended daily time commitment for home exercises was approximately more than 30 min. The instruction manual included methods for gaze stability exercises, postural control, and gait training, along with precautions to prevent falls and guidance on managing severe dizziness induced by the exercises. At each weekly visit, adherence to the prescribed home exercise regimen was reviewed and reinforced. Symptoms and physical findings were assessed at subsequent visits, and the training intensity was modified accordingly.

### 2.7 Statistical analysis

The data were analyzed using IBM SPSS Statistics software (version 28; IBM Corp., Armonk, NY, USA). Continuous variables are presented as means with standard deviation, and categorical variables are described as counts and percentages of patients. The independent-samples t test was used for normally distributed data to compare continuous variables between the two groups, and the Mann–Whitney U test was employed for non-normally distributed data. The ratios of categorical variables among the groups were compared using the Pearson χ2 test or Fisher’s exact test. To compare paired continuous variables, the paired-sample t test was conducted for normally distributed data, and the Wilcoxon signed rank test was conducted for non-normally distributed data. To compare paired categorical variables, the McNemar test was performed. A significance level of *p* < 0.05 was considered statistically significant for all tests.

## 3 Results

The diagnoses for the entire study population are presented in Table 1. The most prevalent condition among the patients was AUVH (*n* = 37), followed by CUVH, BVH, posttraumatic dizziness, Ménière’s disease, presbyvestibulopathy, vestibular schwannoma, persistent postural-perceptual dizziness, vestibular migraine, and vestibular paroxysmia. Overall, 56.7% (51/90) of the study population completed all 4 CVRT sessions, while 16.7% (15/90) dropped out after just one CVRT session. Patients with vestibular migraine showed 100% completion of the 4th session, although this disease category consisted of only two participants. Patients with trauma showed the second highest completion of the 4th session (87.5%), followed by the patients with presbyvestibulopathy and vestibular schwannoma (66.7%, each). The proportion of patients who completed 3 or 4 sessions was 66.7% overall. Among the diagnostic categories, the proportions of adherents were highest for vestibular schwannoma and vestibular migraine (both 100%), though the sample sizes in these subgroups were small. This was followed by trauma (87.5%), and CUVH (86.7%).

**Table 1:** Diagnosis of the study population and completed CVRT sessions.

Diagnosis	Subjects, *n* (%)	Completed CVRT sessions, *n* (%)			
		#1	#2	#3	#4
AUVH	37	6	8	2	21
CUVH	15	1	1	5	8
BVH	13	4	2	1	6
Trauma	8	0	1	0	7
Ménière’s disease	6	2	1	0	3
Presbyvestibulopathy	3	1	0	0	2
Vestibular schwannoma	3	0	0	1	2
PPPD	2	1	1	0	0
Vestibular migraine	2	0	0	0	2
Vestibular paroxysmia	1	0	1	0	0
Total, *n* (%)	90 (100.0)	15 (16.7)	15 (16.7)	9 (10.0)	51 (56.7)

CVRT, customized vestibular rehabilitation therapy; AUVH, acute unilateral vestibular hypofunction; CUVH, chronic unilateral vestibular hypofunction; BVH, bilateral vestibular hypofunction; PPPD, persistent postural-perceptual dizziness.

Table 2 exhibits the initial clinical characteristics of the total study population and comparison between adherents (*n* = 60) and non-adherents (*n* = 30). The comparison of initial characteristics between adherents and non-adherents showed no statistically significant differences. Both groups had similar distributions of sex, age, subjective symptom severity (VVAS, K-DHI, K-ABC), and functional measures such as DVA, and FRT. VFT results (VNG, VHIT, and SOT scores) were also comparable. Average number of falls reported was slightly higher in non-adherents (3.3 ± 3.1) compared to adherents (2.4 ± 2.8), but this difference was not statistically significant.

**Table 2:** Comparison of demographics and initial clinical manifestations.

	Total (*n* = 90)	Adherents (*n* = 60)	Non-adherents (*n* = 30)
Sex, *n* (%)			
Male	45 (50.0%)	31 (51.7%)	14 (46.7%)
Female	45 (50.0%)	29 (48.3%)	16 (53.3%)
Age (years)	59.1 ± 14.4	58.9 ± 15.1	59.6 ± 13.2
VVAS-D	5.2 ± 2.3	5.3 ± 2.2	5.0 ± 2.5
VVAS-O	4.4 ± 2.8	4.6 ± 2.8	4.0 ± 2.8
VVAS-I	5.1 ± 2.6	5.3 ± 2.5	4.8 ± 2.6
K-DHI	50.5 ± 25.9	51.4 ± 24.2	48.8 ± 29.4
K-ABC scale	48.4 ± 26.0	48.3 ± 25.6	48.4 ± 27.2
DVA (lines)	4.8 ± 2.5	4.7 ± 2.4	4.9 ± 2.5
FRT (cm)	25.3 ± 6.8	26.0 ± 7.2	23.9 ± 6.0
VNG			
CP	46.2 ± 30.4	48.6 ± 31.1	41.4 ± 28.7
SN, % (*n*)	45.6% (41)	45.0% (27)	46.7% (14)
VHIT - Abnormal, % (*n*)	40.0% (36)	36.7% (22)	46.7% (14)
SOT			
Composite score	57.5 ± 15.0	59.1 ± 14.4	54.3 ± 15.9
Abnormal ratio, % (*n*)			
Somatosensory	8.9% (8)	6.7% (4)	13.3% (4)
Visual	40.0% (36)	38.3% (23)	43.3% (13)
Vestibular	70.0% (63)	68.3% (41)	73.3% (22)
Visual preference	13.3% (12)	13.3% (8)	13.3% (4)
Falls	2.7 ± 2.9	2.4 ± 2.8	3.3 ± 3.1

VVAS, vestibular visual analog scale; VVAS-D, VVAS dizziness; VVAS-O, VVAS oscillopsia; VVAS-I, VVAS imbalance; K-DHI, Korean dizziness handicap inventory; K-ABC, Korean activities-specific balance confidence; DVA, dynamic visual acuity; FRT, functional reach test; VNG, videonystagmography; CP, caloric paresis (%); SN, spontaneous nystagmus; VHIT, video head impulse test; SOT, sensory organization test.

Table 3 presents a comparative analysis of demographic characteristics, subjective and objective findings between adherents and non-adherents, for each vestibulopathy category (AUVH, CUVH, and BVH). In patients with AUVH, subjective symptoms did not significantly differ between the AUVH-A and AUVH-NA subgroups. However, a significant difference was observed in the FRT scores, where AUVH-A patients exhibited significantly better balance function (27.2 ± 5.5 cm) compared to the AUVH-NA patients (23.3 ± 5.6 cm) (*p* = 0.045). This suggests that patients with better baseline balance function were more likely to adhere to the CVRT. The association between poorer balance function and dropout was observed only in AUVH patients. In the CUVH category, despite the small sample size of CUVH-NA (*n* = 2), there were noticeable differences in subjective dizziness scores including VVAS, K-DHI and K-ABC scores between CUVH-A and CUVH-NA patients. Non-adherent patients consistently reported markedly lower dizziness severity across all subjective measures compared to adherent patients. Notably, the VVAS-D score was significantly lower in CUVH-NA (1.5 ± 0.7) compared to CUVH-A (5.6 ± 1.7) (*p* = 0.019). These findings suggest that CUVH patients with initially milder dizziness symptoms were less likely to adhere to the CVRT. In patients with BVH, there were no significant differences between the adherent and non-adherent subgroups in terms of subjective symptoms or objective clinical measures. VFT results revealed no significant differences between adherents and non-adherents, either in the total study population or within each disease category.

**Table 3:** Comparison of initial clinical parameters between the adherent and non-adherent subgroups.

	AUVH (*n* = 37)			CUVH (*n* = 15)			BVH (*n* = 13)		
	AUVH-A (*n* = 23)	AUVH-NA (*n* = 14)	*p*	CUVH-A (*n* = 13)	CUVH-NA (*n* = 2)	*p*	BVH-A (*n* = 7)	BVH-NA (*n* = 6)	*p*
Sex, *n* (%)			0.898			1			0.266
Male	12 (52.2%)	7 (50.0%)		5 (38.5%)	1 (50.0%)		6 (85.7%)	3 (50.0%)	
Female	11 (47.8%)	7 (50.0%)		8 (61.5%)	1 (50.0%)		1 (14.3%)	3 (50.0%)	
Age (years)	56.3 ± 15.1	60.1 ± 11.6	0.42	58.4 ± 15.5	73.5 ± 5.0	0.148	57.4 ± 14.0	51.0 ± 15.3	0.366
VVAS-D	5.4 ± 2.2	5.0 ± 2.9	0.642	5.6 ± 1.7^§^	1.5 ± 0.7^§^	0.025^§^	5.4 ± 3.3	6.0 ± 1.3	0.695
VVAS-O	4.8 ± 2.9	4.4 ± 3.2	0.648	4.6 ± 2.3	1.5 ± 2.1	0.103	4.9 ± 4.3	5.2 ± 2.2	0.836
VVAS-I	5.3 ± 2.7	4.8 ± 2.9	0.614	5.1 ± 2.3	2.5 ± 3.5	0.199	6.6 ± 2.5	6.0 ± 2.0	0.663
K-DHI	50.1 ± 27.9	48.9 ± 33.8	0.902	51.4 ± 22.9	27.0 ± 18.4	0.232	52.3 ± 19.7	53.3 ± 31.8	0.945
K-ABC scale	49.7 ± 30.0	54.0 ± 23.4	0.653	56.6 ± 24.8	71.0 ± 38.2	0.61	37.4 ± 22.7	30.7 ± 25.7	0.445
DVA (lines)	4.4 ± 2.3	4.9 ± 1.9	0.44	4.8 ± 2.2	5.0 ± 4.2	1	5.2 ± 4.0	6.8 ± 3.2	0.366
FRT (cm)	27.2 ± 5.5^§^	23.3 ± 5.6^§^	0.045^§^	26.5 ± 10.9	27.2 ± 4.0	0.932	27.4 ± 4.7	23.8 ± 4.8	0.198
VNG									
CP	61.9 ± 23.5	61.7 ± 20.8	0.979	63.0 ± 23.3	50.0 ± 29.7	0.734	15.6 ± 9.6	15.5 ± 23.0	0.366
SN, % (*n*)	78.3% (18)	71.4% (10)	0.705	23.1% (3)	50.0% (1)	0.476	14.3% (1)	33.3% (2)	0.559
VHIT - Abnormal, % (*n*)	73.9% (17)	78.6% (11)	1	53.8% (7)	100.0% (2)	0.486	42.9% (3)	66.7% (4)	0.592
SOT	-	-		-	-		-	-	
Composite score	59.0 ± 14.0	54.2 ± 14.0	0.316	61.9 ± 17.4	41.0 ± 1.4	0.126	51.9 ± 13.1	50.2 ± 16.0	0.838
Abnormal ratio, % (*n*)	-	-		-	-		-	-	
Somatosensory	4.3% (1)	14.3% (2)	0.544	7.7% (1)	0.0% (0)	1	14.3% (1)	16.7% (1)	1
Visual	34.8% (8)	35.7% (5)	1	30.8% (4)	100.0% (2)	0.143	71.4% (5)	33.3% (2)	0.286
Vestibular	73.9% (17)	78.6% (11)	1	53.8% (7)	100.0% (2)	0.486	85.7% (6)	83.3% (5)	1
Visual preference	8.7% (2)	7.1% (1)	1	15.4% (2)	0.0% (0)	1	14.3% (1)	50.0% (3)	0.266
Falls	2.4 ± 2.8	3.3 ± 2.9	0.335	2.0 ± 3.1	5.5 ± 0.7	0.15	3.7 ± 2.8	4.0 ± 3.6	0.875

^§^Significant at *p* < 0.05. AUVH, acute unilateral vestibular hypofunction; CUVH, chronic unilateral vestibular hypofunction; BVH, bilateral vestibular hypofunction; A, adherent; NA, non-adherent; VVAS, vestibular visual analog scale; VVAS-D, VVAS dizziness; VVAS-O, VVAS oscillopsia; VVAS-I, VVAS imbalance; K-DHI, Korean dizziness handicap inventory; K-ABC, Korean activities-specific balance confidence; DVA, dynamic visual acuity; FRT, functional reach test; VNG, videonystagmography; CP, caloric paresis (%); SN, spontaneous nystagmus; VHIT, video head impulse test; SOT, sensory organization test.

Table 4 presents the pre- and post-CVRT dizziness-related questionnaire scores and VFT results for patients who completed the assessments within one week after their final therapy session. Across the total study population, significant improvements were observed in all dizziness-related measures, with VVAS-D, VVAS-O, and VVAS-I scores showing marked reductions post-treatment (*p* = 0.002, <0.001, and < 0.001, respectively), indicating a substantial decrease in dizziness, oscillopsia, and imbalance following CVRT. The K-DHI score was also significantly reduced (*p* < 0.001), reflecting a lower perceived handicap related to dizziness, while the K-ABC scale showed significant improvement (*p* < 0.001), indicating enhanced balance confidence. The VOR gain of horizontal canal in VHIT, as well as the composite score of the SOT, ratios of visual dysfunction, vestibular dysfunction, and visual preference, and the number of falls, also showed significant improvements (*p* = <0.001, <0.001, 0.001, <0.001, 0.031, and < 0.001, respectively). In the AUVH category, significant improvements were observed in all dizziness-related measures, with VVAS-D, VVAS-O, and VVAS-I scores showing marked reductions post-treatment (*p* < 0.001), indicating a substantial decrease in dizziness, oscillopsia, and imbalance following CVRT. The K-DHI score and the K-ABC scale also showed significant improvement (*p* = 0.034, and 0.004). The VFT results showed significant improvement in spontaneous nystagmus, VOR gain, composite score, the ratios of visual and vestibular dysfunctions, and the number of falls (*p* = 0.008, <0.001, <0.001, 0.004, <0.001, and 0.003, respectively). However, in the CUVH category, while there was an improvement in the most of post-treatment scores, the changes did not reach statistical significance. The BVH category demonstrated significant improvements in both the K-DHI and K-ABC scales (*p* = 0.020 and *p* = 0.031, respectively), although changes in the VVAS scores and the VFT results were not statistically significant.

**Table 4:** Pre- and post-CVRT dizziness-related questionnaire scores and VFT results.

	Total (*n* = 78)			AUVH (*n* = 33)			CUVH (*n* = 15)			BVH (*n* = 9)		
	Pre-CVRT	Post-CVRT	*P*	Pre-CVRT	Post-CVRT	*P*	Pre-CVRT	Post-CVRT	*P*	Pre-CVRT	Post-CVRT	*P*
VVAS-D	5.0 ± 2.4	4.2 ± 2.7	0.002^§^	5.1 ± 2.5	3.7 ± 3.0	0.001^§^	5.1 ± 2.1	4.6 ± 2.7	0.543	5.8 ± 2.9	4.9 ± 2.0	0.225
VVAS-O	4.4 ± 2.9	3.5 ± 2.7	<0.001^§^	4.6 ± 3.1	2.5 ± 2.4	<0.001^§^	4.2 ± 2.5	3.9 ± 3.3	0.704	4.8 ± 4.0	4.7 ± 2.2	0.944
VVAS-I	5.1 ± 2.6	3.8 ± 2.7	<0.001^§^	5.0 ± 2.8	3.1 ± 2.8	<0.001^§^	4.7 ± 2.5	3.3 ± 2.7	0.094	6.7 ± 2.4	5.1 ± 2.6	0.133
K-DHI	49.8 ± 26.2	41.8 ± 28.1	<0.001^§^	50.0 ± 31.0	39.4 ± 31.8	0.034^§^	48.1 ± 23.4	41.7 ± 30.5	0.128	50.4 ± 17.9	37.8 ± 13.6	0.020^§^
K-ABC	49.8 ± 26.3	61.0 ± 28.7	<0.001^§^	51.0 ± 28.7	66.2 ± 32.1	0.004^§^	58.5 ± 25.6	62.2 ± 26.5	0.432	33.0 ± 21.7	56.3 ± 20.1	0.031^§^
VNG	(*n* = 52)			(*n* = 23)			(*n* = 9)			(*n* = 4)		
SN, % (*n*)	48.1% (25)	4.6% (18)	0.118	82.6% (19)	52.2% (12)	0.008^§^	11.1% (1)	0 (0.0%)	1	25.0% (1)	50.0% (2)	0.375
VHIT – VOR gain	(*n* = 48)			(*n* = 21)			(*n* = 9)			(*n* = 4)		
	0.83 ± 0.21	0.85 ± 0.21	<0.001^§^	0.78 ± 0.17	0.85 ± 0.19	<0.001^§^	0.83 ± 0.22	0.85 ± 0.20	0.26	0.70 ± 0.47	0.62 ± 0.36	0.273
SOT	(*n* = 52)			(*n* = 22)			(*n* = 9)			(*n* = 4)		
Composite score	59.2 ± 14.2	71.2 ± 12.5	<0.001^§^	7.5 ± 15.1	4.7 ± 7.2	<0.001^§^	62.3 ± 17.2	69.4 ± 17.6	0.05	51.5 ± 15.7	54.3 ± 15.3	0.066
Abnormal ratio, % (*n*)												
Somatosensory	7.7% (4)	3.8% (2)	0.625	13.6% (3)	4.5% (1)	0.25	0.0% (0)	11.1% (1)	1	0.0% (0)	0.0% (0)	1
Visual	44.2% (23)	19.2% (52)	0.001^§^	45.5% (10)	9.1% (2)	0.004^§^	33.3% (3)	11.1% (1)	0.5	25.0% (1)	75.0% (3)	0.5
Vestibular	67.3% (35)	28.8% (15)	<0.001^§^	77.3% (17)	18.2% (4)	<0.001^§^	44.4% (4)	33.3% (3)	1	75.0% (3)	75.0% (3)	1
Visual preference	11.5% (6)	0.0% (0)	0.031^§^	4.5% (1)	0.0% (0)	0.5	11.1% (1)	0.0% (0)	1	75.0% (3)	50.0% (2)	0.5
Falls	2.2 ± 2.7	0.7 ± 1.8	<0.001^§^	2.5 ± 3.0	0.1 ± 0.6	0.003^§^	2.0 ± 2.8	1.3 ± 2.6	0.18	4.3 ± 2.9	3.3 ± 2.5	0.18

^§Significant at p < 0.05. CVRT, customized vestibular rehabilitation therapy; VFT, vestibular function test; AUVH, acute unilateral vestibular hypofunction; CUVH, chronic unilateral vestibular hypofunction; BVH, bilateral vestibular hypofunction; VVAS, vestibular visual analog scale; VVAS-D, VVAS dizziness; VVAS-O, VVAS oscillopsia; VVAS-I, VVAS imbalance; K-DHI, Korean dizziness handicap inventory; K-ABC, Korean activities-specific balance confidence; VNG, videonystagmography; SN, spontaneous nystagmus; VHIT, video head impulse test; VOR, vestibuloocular reflex; SOT, sensory organization test.^

Table 5 compares pre- to post-treatment changes in dizziness-related questionnaire scores (*Δ* VVAS, Δ K-DHI, Δ K-ABC, and the ratios of patients with improvements exceeding MCIDs of K-DHI and K-ABC) between adherent and non-adherent patients, both for the total study population and within specific diagnostic categories (AUVH, CUVH, BVH). In all metrics and across all diagnostic categories, there were no statistically significant differences between the adherent and non-adherent subgroups. For the total study population, changes in VVAS-D (0.9 ± 2.5 vs. 0.7 ± 1.9, *p* = 0.819), VVAS-O (0.8 ± 3.1 vs. 1.2 ± 2.8, *p* = 0.952), VVAS-I (1.3 ± 2.5 vs. 1.2 ± 2.0, *p* = 0.885), K-DHI (7.1 ± 18.1 vs. 10.9 ± 31.0, *p* = 0.561), and K-ABC scale (−12.8 ± 29.5 vs. -6.3 ± 21.0, *p* = 0.388) did not differ significantly between the adherents and non-adherents. Likewise, the proportions of patients with improvements exceeding the MCID values on the K-DHI and K-ABC scale were not significantly different between the adherents and non-adherents. This pattern of non-significant differences was similarly observed in each diagnostic category (AUVH, CUVH, BVH).

**Table 5:** Comparison of changes in pre- to post-CVRT dizziness-related questionnaire scores between the adherent and non-adherent patients.

	Total			AUVH			CUVH			BVH		
	Adherents (*n* = 60)	Non-adherents (*n* = 18)	*P*	AUVH-A (*n* = 23)	AUVH-NA (*n* = 10)	*P*	CUVH-A (*n* = 13)	CUVH-NA (*n* = 2)	*P*	BVH-A (*n* = 7)	BVH-NA (*n* = 2)	*P*
Δ VVAS-D^a^	0.9 ± 2.5	0.7 ± 1.9	0.819	1.6 ± 2.3	0.9 ± 2.2	0.417	0.6 ± 3.1	−0.5 ± 0.7	0.476	0.7 ± 2.3	1.5 ± 0.7	0.667
Δ VVAS-O^a^	0.8 ± 3.1	1.2 ± 2.8	0.952	2.1 ± 3.0	2.2 ± 3.3	0.893	0.3 ± 3.6	0.5 ± 0.7	0.686	0.3 ± 3.9	−0.5 ± 3.5	0.889
Δ VVAS-I^a^	1.3 ± 2.5	1.2 ± 2.0	0.885	2.1 ± 2.0	1.5 ± 2.3	0.490	1.5 ± 3.2	1.0 ± 2.8	0.800	1.6 ± 3.1	1.5 ± 2.1	0.889
Δ K-DHI^a^	7.1 ± 18.1	10.9 ± 31.0	0.561	9.9 ± 20.3	12.0 ± 40.6	0.802	6.2 ± 16.0	8.0 ± 17.0	0.933	13.7 ± 14.5	9.0 ± 9.9	0.667
Δ K-DHI ≥ MCID, % (*n*)	23.3% (14)	33.3% (6)	0.539	26.1% (6)	40.0% (4)	0.444	23.1% (3)	50.0% (1)	0.476	42.9% (3)	0.0% (0)	0.500
Δ K-ABC^a^	−12.8 ± 29.5	−6.3 ± 21.0	0.388	−18.3 ± 29.9	−8.0 ± 24.6	0.743	−3.0 ± 13.4	−8.0 ± 25.5	0.933	−26.0 ± 29.6	−14.0 ± 15.6	0.667
Δ K-ABC ≥ MCID, % (*n*)	33.3% (20)	38.9% (7)	0.664	47.8% (11)	50.0% (5)	1.000	7.7% (1)	50.0% (1)	0.257	57.1% (4)	50.0% (1)	1.000

^aThe positive value indicates an improvement in symptoms, while the negative value indicates a worsening of symptoms. CVRT, customized vestibular rehabilitation therapy; AUVH, acute unilateral vestibular hypofunction; CUVH, chronic unilateral vestibular hypofunction; BVH, bilateral vestibular hypofunction; VVAS, visual vestibular analog scale; Δ VVAS-D, pre- to post-CVRT change of VVAS dizziness; Δ VVAS-O pre- to post-CVRT change of VVAS oscillopsia; Δ VVAS-I, pre- to post-CVRT change of VVAS imbalance; Δ K-DHI, pre- to post-CVRT change of Korean dizziness handicap inventory; Δ K-ABC, pre- to post-CVRT change of Korean activities-specific balance confidence; MCID, minimally clinical important difference.^

Table 6 presents the comparison results of questionnaires completed between 1 and 2 weeks after starting the CVRT, between adherents and non-adherents. Since only those who completed mid-treatment surveys were included, the number of patients in each category differs from the initial analysis. In the total study population and the AUVH category, non-adherent patients showed significantly lower scores in all VVAS items and the K-DHI, indicating milder dizziness symptoms. Although the K-ABC scale also showed higher confidence in balance among non-adherent patients, this difference was not statistically significant. Notably, among patients with AUVH, AUVH-NA showed symptom scores reduced to 10–20% of the maximum, indicating only mild residual symptoms. In the CUVH category, non-adherent patients also reported lower VVAS and K-DHI scores, though the small sample size (*n* = 1) for the non-adherents limits the statistical power. In the BVH category, VVAS and K-DHI scores were similar between adherent and non-adherent patients, reflecting a moderate level of symptom severity regardless of treatment adherence. The K-ABC scale showed a noticeably lower confidence in balance for BVH-NA subgroup, although this difference was not statistically significant.

**Table 6:** Comparison of mid-CVRT dizziness-related questionnaire scores between the adherent and non-adherent groups.

	Total			AUVH			CUVH			BVH		
	Group A (*n* = 57)	Group NA (*n* = 11)	*p*	AUVH-A (*n* = 21)	AUVH-NA (*n* = 6)	*p*	CUVH-A (*n* = 13)	CUVH-NA (*n* = 1)	*p*	BVH-A (*n* = 7)	BVH-NA (*n* = 2)	*p*
VVAS-D^a^	5.1 ± 2.4	3.0 ± 2.2	0.010^§^	4.6 ± 2.2	1.7 ± 1.9	0.007^§^	5.2 ± 2.5	3	0.429	4.3 ± 2.2	5.5 ± 0.7	0.500
VVAS-O^a^	4.8 ± 2.6	2.2 ± 1.9	0.002^§^	4.2 ± 2.6	0.8 ± 1.0	0.005^§^	5.0 ± 2.7	2	0.286	5.0 ± 3.6	5.0 ± 0.0	1.000
VVAS-I^a^	5.0 ± 2.4	2.9 ± 2.4	0.010^§^	4.2 ± 2.6	1.3 ± 1.6	0.017^§^	5.1 ± 2.1	2	0.143	5.0 ± 2.8	5.5 ± 0.7	0.667
K-DHI^a^	43.7 ± 25.8	23.3 ± 23.6	0.022^§^	43.7 ± 27.8	18.3 30.9	0.026^§^	48.3 ± 25.0	20	0.429	38.6 ± 10.2	35.0 ± 1.4	0.889
K-ABC^a^	53.8 ± 24.6	65.7 ± 32.7	0.168	56.1 ± 27.7	74.0 ± 37.1	0.207	57.1 ± 24.8	70	0.571	51.0 ± 16.3	31.5 ± 23.3	0.333

^§Significant at p < 0.05. aThe positive value indicates an improvement in symptoms, while the negative value indicates a worsening of symptoms. CVRT, customized vestibular rehabilitation therapy; A, adherent; NA, non-adherent; AUVH, acute unilateral vestibular hypofunction; Δ, pre- to post-CVRT change of dizziness-related questionnaire score; VVAS, vestibular visual analog scale; VVAS-D, VVAS dizziness; VVAS-O, VVAS oscillopsia; VVAS-I, VVAS imbalance; K-ABC, Korean activities-specific balance confidence; K-DHI, Korean dizziness handicap inventory.^

## 4 Discussion

Of the total number of patients in our study, 56.7% completed all four CVRT sessions (Table 1), which differed from previous studies. Yardley and Kirby reported an adherence rate of only 37.5% for VRT, where adherence was defined as completing the recommended exercises for the recommended duration or until asymptomatic (8). Meldrum et al. and Soto-Varela et al. reported 77 and 82.5% adherence rates, respectively (39, 40). However, it is important to note that each study had variations in participant characteristics, frequency, exercise time, duration of treatment, and costs associated with VRT.

Our analysis revealed no significant differences in initial clinical characteristics between the adherent and non-adherent groups within the entire study population (Table 2). Age and sex had no statistically significant impact on adherence to CVRT, even though one might have anticipated a decline in adherence with advancing age, potentially attributed to reduced mobility independence. As shown by the results of the K-ABC, K-DHI, and VVAS questionnaires, patients who adhered to CVRT tended to have more severe subjective symptoms than those who dropped out, but this was not statistically significant. Non-adherent patients displayed less favorable results in postural maintenance-related assessments, including the FRT, SOT composite score, and the number of falls, although these differences were not statistically significant. These findings suggest that the initial characteristics, including subjective symptom severity, functional performance, and vestibular function test results, did not significantly differ between the adherents and the non-adherents.

Since this study was a retrospective study, the cause and the status of the disease were varied, which may explain why there was no significant difference between the adherent and non-adherent groups. Because AUVH, CUVH, and BVH are the major categories of vestibular dysfunction with distinctly different disease characteristics and with a relatively sufficient sample size, we further analyzed the adherence factors in these three categories (Table 3). In our study, AUVH was the most prevalent disease (*n* = 37, 41.1%). Patients with AUVH who discontinued treatment tended to have initially milder dizziness symptoms in questionnaire assessments (i.e., VVAS and K-DHI), but worse postural balance assessments (i.e., FRT and SOT), with a statistically significant difference in FRT (*p* = 0.045). This finding suggests that AUVH patients with better baseline FRT scores were more likely to adhere to CVRT. Given the acute and severe nature of AUVH symptoms, including imbalance, nausea, and vomiting, better FRT performance may reflect less severe balance dysfunction, which could reduce the physical and psychological barriers to completing CVRT. Conversely, poorer FRT scores may indicate greater discomfort and challenges during exercises, discouraging adherence. These findings are consistent with the study by Soto-Varela et al. (40), which reported that patients with poor balance metrics, including center of gravity balancing and limits of stability, were more likely to drop out of therapy. They concluded that patients requiring treatment for balance disorders are at higher risk of abandoning therapy and therefore require careful attention.

Patients with CUVH having initially mild symptoms were less likely to be adherent in our study. Among 15 patients diagnosed as CUVH, the majority (13 patients) demonstrated adherence to CVRT, while only two patients discontinued CVRT. Given the limited number of non-adherent individuals, performing precise statistical comparisons between these two subgroups proved challenging. Nevertheless, CUVH-NA showed remarkably milder symptoms across all VVAS items, with scores averaging less than 3 (1.5 ± 0.7 for VVAS dizziness, 1.5 ± 2.1 for VVAS oscillopsia, and 2.5 ± 3.5 for VVAS imbalance) compared to CUVH-A. In addition, CUVH-NA displayed an average K-ABC scale score of 71.0 ± 38.2 and a K-DHI score of 27.0 ± 18.4, indicative of mild functional impairment (41). The differences in questionnaire scores between the two subgroups were substantial, with a mean difference of 3.5 for VVAS dizziness, 3.1 for VVAS oscillopsia, 2.6 for VVAS imbalance, 14.4 for the K-ABC scale, and 24.4 for K-DHI. A significant difference was observed in the VVAS dizziness scores (*p* = 0.019). This finding suggests that patients with mild dizziness with CUVH are more likely to drop out of CVRT, possibly because of a decreased desire for treatment.

As for patients with BVH (*n* = 13), adherence rates were the lowest among all disease categories, with no significant differences observed between adherent and non-adherent patients in questionnaire scores, physical examination findings, or VFT results. The chronic dizziness and severe balance impairments inherent to BVH likely contributed to these low adherence rates. Furthermore, symptom improvement in BVH typically requires a longer duration than the four-week therapy protocol used in this study, limiting our ability to fully assess treatment effects. Nevertheless, this study emphasizes that BVH patients face significant challenges in adhering even to short-term rehabilitation programs, underscoring the need for clinicians to implement strategies that enhance adherence and sustain long-term engagement.

As indicated in Table 4, subjective symptoms improved significantly after CVRT in the total cohort. When analyzed by diagnostic category, AUVH patients showed statistically significant improvements across all measures, including the VVAS, K-DHI, and K-ABC scales, suggesting marked symptom reduction following CVRT. In contrast, CUVH patients also demonstrated improvements across all measures, although these changes did not reach statistical significance. For BVH patients, significant improvements were observed in the K-DHI and K-ABC scales, indicating enhanced functional and balance confidence, although other subjective symptoms (measured by the VVAS) remained unchanged. An analysis of VFT results revealed significant improvements in VOR gain and SOT parameters across the total study population. Notably, these improvements were most prominent in AUVH patients, who also showed significant improvement in spontaneous nystagmus, as expected. However, no marked improvements in VFT parameters were observed in CUVH and BVH patients. These findings suggest that while CVRT is broadly effective in alleviating dizziness-related symptoms, the extent of improvement may vary according to vestibular disorder subtype, with AUVH patients showing the most substantial gains and patients with CUVH and BVH experiencing more selective improvements in certain domains. The limited duration of therapy may also have contributed to these differences, as extended treatment is often required for chronic vestibulopathy (33).

It was expected that patients adherent to CVRT would experience a greater improvement in symptoms after CVRT, but the change in symptoms assessed by the dizziness-related questionnaire after CVRT was similar between the adherent and non-adherent patients (Table 5). This result suggests that adherence to CVRT did not significantly affect the post-treatment improvements in dizziness-related symptoms. Similarly, a previous study also reported no significant link between the efficacy of CVRT and adherence, with suggesting that patients who experience improvements too early might be less motivated to strictly follow the prescribed exercise regimen (15).

Several hypotheses can explain why patients who adhered to 3–4 sessions of CVRT and those who discontinued after only 1–2 sessions demonstrated similar levels of symptom improvement: (1) Spontaneous recovery: Some patients may have experienced rapid natural recovery, particularly those with acute conditions like AUVH, leading them to discontinue therapy early as symptoms resolved without needing further intervention. (2) Rapid treatment response: Some patients might experience significant symptom relief within 1–2 sessions, leading them to perceive further treatment as unnecessary. (3) Mild initial symptoms: Patients who began treatment with milder dizziness symptoms may have recovered more quickly or lacked the motivation to continue therapy. This is likely the case in CUVH, where patients with less severe symptoms may discontinue therapy early. (4) Physical limitations and chronic conditions: In patients with moderate-to-severe dizziness, postural instability, or gait disturbances—particularly in chronic conditions like BVH—improvement may not be evident after only 1–2 sessions. Such patients may face difficulties attending treatment sessions due to their physical condition or may feel that therapy is not effective, leading to early discontinuation.

As this study is retrospective in nature, it is not possible to directly test these hypotheses through our dataset. However, to explore the potential influence of rapid symptom improvement (Hypotheses 2 and 3), we analyzed the early-CVRT symptom change from patients who completed assessments at 1–2 weeks after starting the CVRT (Table 6). In the total study population and AUVH category, non-adherent patients demonstrated significantly lower scores on all VVAS items and K-DHI on the mid-treatment questionnaires, suggesting that their dizziness symptoms were milder than those of adherent patients. Although non-adherent patients also reported higher K-ABC scores (indicating greater balance confidence), this difference was not statistically significant. Particularly in AUVH, non-adherent patients had symptom scores reduced to 10–20% of the maximum, indicating mild symptoms during the mid-treatment period. These results suggest that patients whose symptoms improved to a mild level early in the treatment were less adherent to the CVRT, a trend specifically observed in AUVH category. In contrast, in BVH patients, symptom severity as measured by VVAS and K-DHI was similar between the BVH-A and the BVH-NA subgroups. However, the K-ABC scores of the BVH-NA patients were lower than BVH-A patients, although not statistically significant. This finding suggests that reduced confidence in postural stability may contribute to challenges in attending therapy sessions, potentially leading to non-adherence in the BVH group. Additionally, the slower pace of symptom improvement in BVH could diminish the perception of treatment efficacy, leading to early dropout.

In summary, the early-treatment analysis highlights that early discontinuation of therapy may be driven by different factors depending on the underlying condition. AUVH patients may discontinue due to rapid symptom improvement, while CUVH patients with initially mild symptoms may not feel the need to continue therapy. In BVH, discontinuation is likely due to a combination of low confidence in balance and slower perceived symptom improvement.

Previous studies have explored the factors influencing adherence to VRT. One study found that lack of understanding of VRT and fear of worsening symptoms during exercise could reduce adherence (42). Factors that discouraged participation in home exercise included an immediate lack of improvement in symptoms after exercise, boredom associated with the exercises, discomfort provoked during exercise, and lack of time (42, 43). Yardley et al. reported a higher adherence to VRT when subjects received telephone consultations that provided VRT goals, encouraged exercise, and offered solutions to problems, compared to when only a booklet about VRT was provided to the subjects (7). Pavlou et al. also found that supervised subjects demonstrated higher adherence compared to unsupervised subjects, but psychological symptoms, such as depression, anxiety, or a history of migraines, did not affect dropout rates (44). A study using a mixed deductive-inductive qualitative approach identified key barriers to adherence among patients in vestibular home exercise programs, including temporary worsening of symptoms due to the exercises, the perception that the exercises were no longer needed as symptoms improved, and limited time availability (43). A study on adherence to a general exercise program among the elderly revealed that higher adherence was associated with fewer chronic diseases, lower Mini-Mental Status Examination scores, lower body mass index, higher socioeconomic status, and higher education level (45).

So far, various attempts have been made to improve the efficacy and adherence of VRT. Geraghty et al. conducted a study on internet-based VRT and found that the group receiving this intervention had significantly lower levels of severity of dizziness and related disability than the usual-care group (46). Meldrum et al. explored virtual reality-based VRT for patients with unilateral peripheral vestibular loss and found no significant differences in physical outcomes and adherence between patients who underwent conventional VRT or virtual reality-based VRT. However, the latter group reported more enjoyment, less difficulty, and less tiredness during the exercises (39). Another study analyzed a smartphone application with a wearable head sensor for VRT, which demonstrated a significant improvement in symptoms after VRT using the application, with an average adherence rate of 30.3% (47). Recently, Hall et al. developed a remote therapeutic monitoring VRT platform application that integrates gaming elements aimed at enhancing adherence. Although adherence levels did not significantly differ from those of the standard VRT group, 68.8% of patients indicated that the gaming format could contribute to improving adherence (48). Another recent study that created an application combining home exercises with gaming revealed that more than 90% of patients reported feeling motivated by the games and trophy rewards (49).

Tailoring the CVRT strategies based on the patients’ symptom characteristics and disease categories may improve their adherence to CVRT and the treatment outcomes. It could be effective to offer more detailed explanations to patients with AUVH who have pronounced balance impairment about the importance and effectiveness of vestibular rehabilitation and starting exercises at lower levels of difficulty to encourage their participation in treatment. For patients with CUVH with mild symptoms, it may be efficient to initially prescribe exercises only once or twice, with short-term follow-ups to discuss the continuation of treatment. Given that patients with BVH experience severe balance impairment, it could be advantageous for individuals facing difficulties in regular hospital visits for CVRT to receive intensive treatment through hospitalization. To optimize treatment results, it is prudent not only to tailor exercise methods and intensity according to the patients’ condition but also to employ diverse strategies to enhance adherence, considering the patients’ specific etiology and clinical profile. For patients expected to show low adherence, scheduling treatment sessions on a per-visit basis from the outset could also be considered.

This study has several limitations. It is a retrospective study, which introduces the possibility of selection bias. As our institution recently initiated the prescription and implementation of CVRT, the number of subjects included in the study was relatively limited. Given the relatively small sample size, corrections for multiple statistical analyses were not performed in this study as they could increase the risk of type II errors (50). Additionally, the study included only patients prescribed four sessions, even though individuals with chronic vestibular hypofunction typically require a rehabilitation period exceeding four weeks. Furthermore, in the analysis of early-CVRT and post-CVRT questionnaire scores, only patients with at least one follow-up visit were included, which leads to potential bias.

To the best of our knowledge, however, this study is the only one that has statistically analyzed factors influencing adherence to CVRT. Identifying and addressing the factors that affect adherence can optimize the treatment strategy by increasing patient participation, thus saving health-care resources. Although not covered in this study, it would be valuable to investigate the impact on adherence of the socioeconomic status of the patients, the educational background, and the presence of a family member living with them. Consistent engagement in rehabilitation exercises at home is also essential for achieving the best treatment outcomes, and the recommended frequency and duration differ depending on the condition. The Clinical Practice Guideline suggests performing gaze stabilization exercises at home 3 times a day for a total of ≥12 min for acute/subacute unilateral vestibular hypofunction, 3–5 times a day for ≥20 min daily for 4–6 weeks for CUVH, and 3–5 times a day for 20–40 min daily for 5–7 weeks for BVH (33). Consequently, future studies investigating how well patients adhere to the prescribed duration, frequency, and techniques of home exercises would be valuable.

## 5 Conclusion

Patients with AUVH showed lower adherence to CVRT when they had worse initial FRT score, and when they experienced rapid improvement of symptoms to the mild degree in the early stages of the treatment. Among vestibulopathy categories, patients with CUVH exhibited the highest adherence rates, while in these CUVH cases, patients with initially milder symptoms were less compliant with CVRT. However, the limited number of participants restricts conclusive analysis, underscoring the need for future studies with a larger patient sample for more robust findings. Conversely, patients with BVH exhibited lower adherence than patients with other common vestibular disorders, which potentially stemmed from their reduced self-confidence in performing everyday activities. Strategic approaches to prescribing CVRT tailored to each patient’s specific etiology and clinical profiles to promote better adherence would be beneficial.

## Data Availability

The raw data supporting the conclusions of this article will be made available by the authors, without undue reservation.

## References

[1] Eleftheriadou A Skalidi N Velegrakis GA. Vestibular rehabilitation strategies and factors that affect the outcome. Eur Arch Otorrinolaringol. (2012) 269:2309–16. doi: 10.1007/s00405-012-2019-2, PMID: 22526580

[2] Nada EH Ibraheem OA Hassaan MR. Vestibular rehabilitation therapy outcomes in patients with persistent postural-perceptual dizziness. Ann Otol Rhinol Laryngol. (2019) 128:323–9. doi: 10.1177/0003489418823017, PMID: 30607985

[3] Acarer A Karapolat H Celebisoy N Ozgen G Colakoglu Z. Is customized vestibular rehabilitation effective in patients with Parkinson’s? NeuroRehabilitation. (2015) 37:255–62. doi: 10.3233/NRE-151258, PMID: 26484517

[4] Han BI Song HS Kim JS. Vestibular rehabilitation therapy: review of indications, mechanisms, and key exercises. J Clin Neurol. (2011) 7:184–96. doi: 10.3988/jcn.2011.7.4.184, PMID: 22259614 PMC3259492

[5] Sulway S Whitney SL. Advances in vestibular rehabilitation. Adv Otorhinolaryngol. (2019) 82:164–9. doi: 10.1159/000490285 30947180

[6] Meldrum D Jahn K. Gaze stabilisation exercises in vestibular rehabilitation: review of the evidence and recent clinical advances. J Neurol. (2019) 266:11–8. doi: 10.1007/s00415-019-09459-x, PMID: 31385017

[7] Yardley L Barker F Muller I Turner D Kirby S Mullee M. Clinical and cost-effectiveness of booklet based vestibular rehabilitation for chronic dizziness in primary care: a single-blind, parallel-group, pragmatic, randomised controlled trial. BMJ. (2012) 344:e2217. doi: 10.1136/bmj.e2237, PMID: 22674920 PMC3368486

[8] Yardley L Kirby S. Evaluation of booklet-based self-management of symptoms in Ménière disease: a randomized controlled trial. Psychosom Med. (2006) 68:762–9. doi: 10.1097/01.psy.0000232269.17906.92, PMID: 17012531

[9] van Vugt VA van der Wouden JC Bosmans JE Smalbrugge M van Diest W Essery R. Guided and unguided in-ternet-based vestibular rehabilitation versus usual care for dizzy adults of 50 years and older: a protocol for a three-armed randomised trial. BMJ Open. (2017) 7:e015479. doi: 10.1136/bmjopen-2016-015479, PMID: 28110290 PMC5253547

[10] Xie M Zhou K Patro N Chan T Levin M Gupta MK. Virtual reality for vestibular rehabilitation: a systematic review. Otol Neurotol. (2021) 42:967–77. doi: 10.1097/MAO.0000000000003155, PMID: 33782257

[11] Nehrujee A Vasanthan L Lepcha A Balasubramanian S. Smartphone-based gaming system for vestibular rehabilitation: a usability study. J Vestib Res. (2019) 29:147–60. doi: 10.3233/VES-190660, PMID: 31356221

[12] van Vugt VA van der Wouden JC Essery R Yardley L Twisk JWR van der Horst HE. Internet-based vestibular rehabilitation with and without physiotherapy support for adults aged 50 and older with a chronic vestibular syndrome in general practice: three armed randomised controlled trial. BMJ. (2019) 367:l5922. doi: 10.1136/bmj.l5922, PMID: 31690561 PMC6829201

[13] Lacour M Bernard-Demanze L. Interaction between vestibular compensation mechanisms and vestibular rehabilitation therapy: 10 recommendations for optimal functional recovery. Front Neurol. (2015) 5:285. doi: 10.3389/fneur.2014.00285, PMID: 25610424 PMC4285093

[14] Giray M Kirazli Y Karapolat H Celebisoy N Bilgen C Kirazli T. Short-term effects of vestibular rehabilitation in patients with chronic unilateral vestibular dys-function: a randomized controlled study. Arch Phys Med Rehabil. (2009) 90:1325–31. doi: 10.1016/j.apmr.2009.01.032, PMID: 19651266

[15] Kim MK Yun SY Lee S Lee JO Sung SY Lee JY. Efficacy of vestibular rehabilitation and its facilitating and hindering factors from real-world clinical data. Front Neurol. (2024) 15:1329418. doi: 10.3389/fneur.2024.1329418, PMID: 38487329 PMC10938910

[16] Se To PL Singh DKA Whitney SL. Effects of customized vestibular rehabilitation plus canalith repositioning maneuver on gait and balance in adults with benign paroxysmal positional Vertigo: a randomized controlled trial. J Vestib Res. (2022) 32:79–86. doi: 10.3233/VES-190731, PMID: 34151874

[17] Nishino LK Ganança CF Manso A de Campos CA Korn CP. Personalized vestibular rehabilitation: medical chart survey with patients seen at the ambulatory of otoneurology of I.S.C.M.S.P. Braz J Otorhinolaryngol. (2005) 71:440–7. doi: 10.1016/s1808-8694(15)31196-4, PMID: 16446957 PMC9441992

[18] Herdman SJ Clendaniel RA. Vestibular Rehabilitation. 4th ed. Philadelphia: F.A. Davis (2014).

[19] Jacobson GP Newman CW. The development of the dizziness handicap inventory. Arch Otolaryngol Head Neck Surg. (1990) 116:424–7. doi: 10.1001/archotol.1990.01870040046011, PMID: 2317323

[20] Han GC Lee EJ Lee JH Park SN Lee HY Jeon EJ. The study of standardization for a Korean adaptation of self-report measures of dizziness. J Korean Bal Soc. (2004) 3:307–25.

[21] Powell LE Myers AM. The activities-specific balance confidence (ABC) scale. J Gerontol A Biol Sci Med Sci. (1995) 50A:M28–34. doi: 10.1093/gerona/50a.1.m28, PMID: 7814786

[22] Myers AM Powell LE Maki BE Holliday PJ Brawley LR Sherk W. Psychological indicators of balance confidence: relationship to actual and perceived abilities. J Gerontol A Biol Sci Med Sci. (1996) 51:M37–43. doi: 10.1093/gerona/51a.1.m37, PMID: 8548512

[23] Wellons RD Duhe SE MacDowell SG Hodge A Oxborough S Levitzky EE. Estimating the minimal clinically important difference for balance and gait outcome measures in individuals with vestibular disorders. J Vestib Res. (2022) 32:223–33. doi: 10.3233/VES-201630, PMID: 35147571

[24] Dankova M Jerabek J Jester DJ Zumrova A Paulasova Schwabova J Cerny R. Clinical dynamic visual acuity in patients with cere-bellar ataxia and vestibulopathy. PLoS One. (2021) 16:e0255299. doi: 10.1371/journal.pone.0255299, PMID: 34324564 PMC8320895

[25] Omaña H Bezaire K Brady K Davies J Louwagie N Power S. Functional reach test, single-leg stance test, and Tinetti performance-oriented mobility assessment for the prediction of falls in older adults: a systematic review. Phys Ther. (2021) 101:1–18. doi: 10.1093/ptj/pzab173, PMID: 34244801

[26] Jongkees LB Philipszoon AJ. Electronystagmography. Acta Otolaryngol Suppl. (1964) 189:1. PMID: 14151845

[27] Strupp M Bisdorff A Furman J Hornibrook J Jahn K Maire R. Acute unilateral vestibulopathy/vestibular neuritis: Diagnostic criteria. J Vestib Res. (2022) 32:389–406. doi: 10.3233/VES-220201, PMID: 35723133 PMC9661346

[28] Strupp M Kim JS Murofushi T Straumann D Jen JC Rosengren SM. Bilateral vestibulopathy: diagnostic criteria consensus doc-ument of the classification Committee of the Bárány Society. J Vestib Res. (2017) 27:177–89. doi: 10.3233/VES-170619, PMID: 29081426 PMC9249284

[29] Macdougall HG McGarvie LA Halmagyi GM Curthoys IS Weber KP. The video head impulse test (vHIT) detects vertical semicircular canal dysfunction. PLoS One. (2013) 8:e61488. doi: 10.1371/journal.pone.0061488, PMID: 23630593 PMC3632590

[30] Strupp M Grimberg J Teufel J Laurell G Kingma H Grill E. Worldwide survey on laboratory testing of vestibular function. Neurol Clin Pract. (2020) 10:379–87. doi: 10.1212/CPJ.0000000000000744, PMID: 33299665 PMC7717631

[31] Yeh JR Hsu LC Lin C Chang FL Lo MT. Nonlinear analysis of sensory organization test for subjects with unilateral vestibular dysfunction. PLoS One. (2014) 9:e91230. doi: 10.1371/journal.pone.0091230, PMID: 24632582 PMC3954723

[32] El-Kashlan HK Shepard NT Asher AM Smith-Wheelock M Telian SA. Evaluation of clinical measures of equilibrium. Laryngoscope. (1998) 108:311–9. doi: 10.1097/00005537-199803000-00002, PMID: 9504600

[33] Hall CD Herdman SJ Whitney SL Anson ER Carender WJ Hoppes CW. Vestibular rehabilitation for peripheral vestibular hypofunction: an updated clinical practice guideline from the academy of neurologic physical therapy of the American Physical Therapy Association. J Neurol Phys Ther. (2022) 46:118–77. doi: 10.1097/NPT.0000000000000382, PMID: 34864777 PMC8920012

[34] Lopez-Escamez JA Carey J Chung WH Goebel JA Magnusson M Mandalà M. Diagnostic criteria for Menière's disease. J Vestib Res. (2015) 25:1–7. doi: 10.3233/VES-150549, PMID: 25882471

[35] Agrawal Y Van de Berg R Wuyts F Walther L Magnusson M Oh E. Presbyvestibulopathy: diagnostic criteria consensus document of the classification committee of the Bárány society. J Vestib Res. (2019) 29:161–70. doi: 10.3233/VES-190672, PMID: 31306146 PMC9249286

[36] Staab JP Eckhardt-Henn A Horii A Jacob R Strupp M Brandt T. Diagnostic criteria for persistent postural-perceptual dizziness (PPPD): consensus document of the committee for the classification of vestibular disorders of the Bárány society. J Vestib Res. (2017) 27:191–208. doi: 10.3233/VES-170622, PMID: 29036855 PMC9249299

[37] Strupp M Lopez-Escamez JA Kim JS Straumann D Jen JC Carey J. Vestibular paroxysmia: Diagnostic criteria. J Vestib Res. (2016) 26:409–15. doi: 10.3233/VES-160589, PMID: 28262641 PMC9249278

[38] Lempert T Olesen J Furman J Waterston J Seemungal B Carey J. Vestibular migraine: Diagnostic Criteria1. J Vestib Res. (2022) 32:1–6. doi: 10.3233/VES-201644, PMID: 34719447 PMC9249276

[39] Meldrum D Herdman S Vance R Murray D Malone K Duffy D. Effectiveness of conventional versus virtual reality-based balance exercises in vestibular rehabilitation for unilateral peripheral vestibular loss: results of a randomized controlled trial. Arch Phys Med Rehabil. (2015) 96:1319–28.e1. doi: 10.1016/j.apmr.2015.02.032, PMID: 25842051

[40] Soto-Varela A Faraldo-García A Del-Río-Valeiras M Rossi-Izquierdo M Vaamonde-Sánchez-Andrade I Gayoso-Diz P. Adherence of older people with instability in vestibular rehabilitation programmes: prediction criteria. J Laryngol Otol. (2017) 131:232–8. doi: 10.1017/S0022215116009932, PMID: 28088930

[41] Whitney SL Wrisley DM Brown KE Furman JM. Is perception of handicap related to functional performance in persons with vestibular dysfunction? Otol Neurotol. (2004) 25:139–43. doi: 10.1097/00129492-200403000-00010 15021773

[42] Dsilva LJ Skop KM Pickle NT Marschner K Zehnbauer TP Rossi M. Use of stakeholder feedback to develop an app for vestibular rehabilitation-input from clinicians and healthy older adults. Front Neurol. (2022) 13:836571. doi: 10.3389/fneur.2022.836571, PMID: 35280295 PMC8907890

[43] Kalderon L Kaplan A Wolfovitz A Levy-Tzedek S Gimmon Y. Barriers and facilitators of vestibular rehabilitation: patients and Physiotherapists' perspectives. J Neurol Phys Ther. (2024) 48:140–50. doi: 10.1097/NPT.0000000000000470, PMID: 38426842 PMC11208053

[44] Pavlou M Bronstein AM Davies RA. Randomized trial of supervised versus unsupervised optokinetic exercise in persons with peripheral vestibular disorders. Neurorehabil Neural Repair. (2013) 27:208–18. doi: 10.1177/1545968312461715, PMID: 23077146

[45] Picorelli AM Pereira LS Pereira DS Felício D Sherrington C. Adherence to exercise programs for older people is influenced by program characteristics and personal factors: a systematic review. J Physiother. (2014) 60:151–6. doi: 10.1016/j.jphys.2014.06.012, PMID: 25092418

[46] Geraghty AWA Essery R Kirby S Stuart B Turner D Little P. Internet-based vestibular rehabilitation for older adults with chronic dizziness: a randomized controlled trial in primary care. Ann Fam Med. (2017) 15:209–16. doi: 10.1370/afm.2070, PMID: 28483885 PMC5422081

[47] Meldrum D Murray D Vance R Coleman S McConnell S Hardiman O. Toward a digital health intervention for vestibular rehabilitation: usability and subjective outcomes of a novel platform. Front Neurol. (2022) 13:836796. doi: 10.3389/fneur.2022.836796, PMID: 35422750 PMC9001890

[48] Hall CD Flynn S Clendaniel RA Roberts DC Stressman KD Pu W. Remote assessment and management of patients with dizziness: development, validation, and feasibility of a gamified vestibular rehabilitation therapy platform. Front Neurol. (2024) 15:1367582. doi: 10.3389/fneur.2024.1367582, PMID: 38872821 PMC11169667

[49] D’Silva LJ Phongsavath T Partington K Pickle NT Marschner K Zehnbauer TP. A gaming app developed for vestibular rehabilitation improves the accuracy of performance and engagement with exercises. Front Med. (2023) 10:1269874. doi: 10.3389/fmed.2023.1269874, PMID: 38076248 PMC10704144

[50] Feise RJ. Do multiple outcome measures require p-value adjustment? BMC Med Res Methodol. (2002) 2:8. doi: 10.1186/1471-2288-2-8, PMID: 12069695 PMC117123

